# Integrative bioinformatics analysis of transcriptional regulatory programs in breast cancer cells

**DOI:** 10.1186/1471-2105-9-404

**Published:** 2008-09-29

**Authors:** Atsushi Niida, Andrew D Smith, Seiya Imoto, Shuichi Tsutsumi, Hiroyuki Aburatani, Michael Q Zhang, Tetsu Akiyama

**Affiliations:** 1 Laboratory of Molecular and Genetic Information, Institute of Molecular and Cellular Biosciences, The University of Tokyo, 1-1-1, Yayoi, Bunkyo-ku, Tokyo, 110-0032, Japan; 2 Cold Spring Harbor Laboratory, Cold Spring Harbor, NY 11274, USA; 3 The Institute of Medical Science, The University of Tokyo, 4-6-1 Shirokanedai, Minato-ku, Tokyo 108-8639, Japan; 4 Genome Science Division, Research Center for Advanced Science and Technology, The University of Tokyo, 4-6-1 Komaba, Meguro, Tokyo, 153-8904, Japan

## Abstract

**Background:**

Microarray technology has unveiled transcriptomic differences among tumors of various phenotypes, and, especially, brought great progress in molecular understanding of phenotypic diversity of breast tumors. However, compared with the massive knowledge about the transcriptome, we have surprisingly little knowledge about regulatory mechanisms underling transcriptomic diversity.

**Results:**

To gain insights into the transcriptional programs that drive tumor progression, we integrated regulatory sequence data and expression profiles of breast cancer into a Bayesian Network, and searched for *cis*-regulatory motifs statistically associated with given histological grades and prognosis. Our analysis found that motifs bound by ELK1, E2F, NRF1 and NFY are potential regulatory motifs that positively correlate with malignant progression of breast cancer.

**Conclusion:**

The results suggest that these 4 motifs are principal regulatory motifs driving malignant progression of breast cancer. Our method offers a more concise description about transcriptome diversity among breast tumors with different clinical phenotypes.

## Background

Deregulation of transcriptional programs leads to development and progression of cancer, and many transcription factors (TFs) have been identified as oncogenes or tumor suppressor genes [1]. In the last decade, microarray technology has revolutionized cancer biology: microarray-based expression profiling studies have revealed that transcriptomes of cancer cells drastically change during carcinogenesis, and vary among different types of tumors.

Among many types of cancers, breast cancer has been attracting numerous investigators armed with microarray technology. Human breast tumors are diverse in their histology, prognosis, and responsiveness to treatments. Microarray technology has unveiled transcriptomic differences among tumors of various phenotypes, and brought great progress in molecular understanding of the phenotypic diversity. For example, Perou *et al*. [2] and Sorlie *et al*. [3] established that breast tumors are classified into five different phenotypic subtypes. van't Veer *et al*. [4] and van de Vijver *et al*. [5] accurately divided breast cancer patients into two groups with favorable or unfavorable outcome, suggesting the potential of microarrays as a diagnostic test to select patients who would need adjuvant therapies. Many other studies have also identified gene signatures that enable us to predict distant metastasis or survival [6-8]. However, compared with the massive knowledge about the transcriptome, we have surprisingly little knowledge about regulatory mechanisms underling transcriptomic diversity.

To analyze the transcriptional regulatory programs, computational approaches that integrate regulatory sequence data with global expression profiles are essential. So far, many approaches have been developed and successfully applied to lower organisms like yeast. For finding motifs that regulate gene expressions in yeast, linear regression-based methods use the correlation between the presence of cis-regulatory motifs and expression values [9,10]. A method employing multivariate adaptive regression spline (MARS) algorithm captured synergistic interactions between regulatory motifs and improved the prediction significantly as compared to that by the linear regression [11]. A method based on Bayesian networks also successfully identified combinational gene regulation by multiple motifs in yeast promoter sequences [12]. On the other hand, such challenges for gene regulation in higher eukaryotes like human are much harder owing to intrinsic complexity of their regulatory systems, and have just started [13,14]. As for breast cancer, although a small number of studies have also tried to decode transcriptional programs in cancer cell [15,16], it also remains to be tested whether transcriptional programs exist that are associated with, and potentially drive, breast tumor malignancy.

In this study, we propose a new approach to decipher transcriptional programs from cancer microarray data. Our method searches for the most probable motif combination associated with clinical phenotypes such as histological grade or survival time. Our approach has two major novel features. First, extending a previous work [12], we introduce a Bayesian scoring function which can treat continuous expression values. Secondly, instead of using raw expression values, we define a "meta-expression value" based on a correlation between gene expression profiles of a gene and a clinical phenotype, and then search for motifs correlated with meta-expression values. We show that application of our method to breast cancer microarray data successfully identified *cis*-regulatory motifs which are associated with malignancy of breast cancer.

## Methods

### Methods Overview

**Figure 1:**
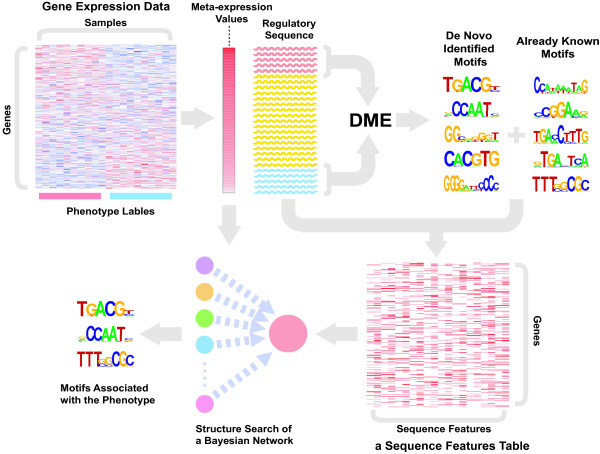
**Schema of our method**. We first calculate correlations between phenotypes and expression values as meta-expression values, while preparing a sequence feature table by searching promoter sequences for *cis*-regulatory motifs. *Cis*-regulatory motif data are prepared from two different sources: already known motifs, which are downloaded from databases, and *de novo *identified motifs, which were discovered by an *ab initio *motif finder program, DME. Then, associations between sequence features and meta-expression values were inferred by structure learning of Bayesian networks.

To elucidate transcriptional programs in cancer cells, we used a bioinformatics method based on Bayesian networks. We integrated regulatory sequences and global expression profiling data, and searched for *cis*-regulatory motifs statistically associated with clinical annotation accompanying the expression profiling data (Fig. 1).

We prepared three types of data to be integrated: regulatory sequences, regulatory motifs and expression profiling data. For regulatory sequences, we used core promoter sequences spanning 500 bp upstream and 100 bp down stream of the transcriptional start sites (TSSs). The regulatory motif data were prepared as position weight matrices (PWMs) by the following method: the known TF binding motifs were obtained from the TRANSFAC [17] and JASPAR database [18]. In addition, to complement missing information of the databases, we obtained potentially novel PWMs using an *ab initio *motif finder program, Discriminating Matrix Enumerator (DME, Smith *et al*., 2005). Among similar types of motif finder programs, an exceptional feature of DME is that it identifies motifs based on relative over-representation between two sets of sequences. To obtain the *de novo *identified motif set, DME was applied to the regulatory sequences of gene groups which display highest and lowest expression values in expression value data. After reducing redundancy of these two PWM sets by clustering, the regulatory sequence of every gene was scored by each PWM. Then, the obtained scores are binarized using multiple thresholds to produce sequence features. Here, each sequence feature indicates the presence of a motif assuming one version of the multiple PWM thresholds. Prepared sequence features are collected to produce a sequence feature table. The sequence feature table is a binary matrix with its rows for genes and its columns for sequence features.

For expression value data, we prepared a publicly available data set of breast cancer expression profiles [7]. The data set includes expression values of 16,425 genes in 252 samples and information about a phenotype of each sample including its histological grades and patient prognosis. In our analyses, in stead of using the raw expression values, we used a "meta-expression value" calculated as a kind of correlation of the raw expression values with the phenotypes (e.g. differential expression between two sample groups of different histological grades or correlation with prognosis). Hence, the expression value matrix is transformed to a vector whose element is a meta-expression value of a gene. The expression value data were divided into training data and test data with a ratio of 3:1. Only information from the training data was used in a series of searches including *de novo *motif search using DME, and the test data were used for statistical evaluation of the result.

To infer associations between sequence features and the meta-expression values, our method learns parents of a single child node with methods originating from Bayesian network leaning. We assumed a two-layer network structure where sequence features regulate the meta-expression values. In this case, the structural learning indicates that the method identifies the subset of sequence features that regulates the meta-expression values of each gene. This probabilistic approach is motivated by the work of Beer and Tavazoie [12], which successfully predicted gene expression patterns from combinations of regulatory motifs in yeast. This approach can analyze nonlinear synergistic effects between regulatory motifs, which are thought to be more critical for gene regulation in higher eukaryotes. It can also incorporate flexible conditions of sequence features, such as the threshold value for PWM search. In the work of Beer and Tavazoie [12], the expression values were binarized to indicate whether each gene is assigned to an expression cluster. However, it is known that such discretization of data leads to loss of information [19]. Moreover, results yielded are potentially dependent on the threshold chosen in the discretization [20]. To solve this problem, our analysis introduces a new scoring function, which can deal with continuous meta-expression values. When a binary sequence feature table and continuous meta-expression value data are given as the input data, the scoring function represents the posterior probability of a model that represents the dependency of the expression values and a combination of sequence features. By a greedy strategy, we searched for the most probable combination of sequence features so as to maximize the scoring function. Starting from the empty model, we iteratively added a sequence feature to the model as long as the value of the scoring function increases.

### Regulatory sequence analysis

For regulatory sequence data, we prepared promoter data of 31,718 human genes from the Ensembl database (Release 40). Additionally, we also retrieved 27,967 mouse promoter sequences for comparative analysis (see below). Assuming the TSS as the start base of the gene assigned in Ensembl, a repeat-masked promoter sequence covering the 500 bp upstream and the 100 bp downstream of the TSS for each gene was extracted from the genome sequences.

For regulatory motif data, we prepared PWMs. The value *f*_*ib *_of a PWM represents frequency of nucleotide base *b *at the *i*-th position in a motif. The frequencies of bases in each position are normalized so that ∑_*b *∈ {*a*, *t*, *g*, *c*} _*f*_*ib *_= 1. If *f*_*ib *_= 0, we assigned *f*_*ib *_= 0.001 to avoid errors in log calculations. We acquired a total of 495 PWMs, which consist of vertebrate 367 PWMs annotated as "good" in TRANSFAC 10.1 [17], 123 PWMs from JASPAR core [18], and 5 PWMs from existing literature [21,22]. We then removed extremely simple or complex PWMs based on their information contents, and made a set of total 449 PWMs. Using the partition around medoids algorithm with the dissimilarity criterion based on the Kullback-Leibler divergence, the 449 PWMs are divided into 250 clusters (see Additional file 1). In the following analyses, we used 250 medoids of the clusters as the already known PWMs

In addition to the already known PWM set, we prepared motifs appearing frequently in promoter sequences of genes with high or low values in the expression value data. For the top 500 and the bottom 500 genes for expression values in the training data, we obtained their promoter sequences (the 500 bp upstream and the 100 bp downstream of the TSS) and those of their mouse homologs. We then searched for motifs relatively overrepresented in either set of sequences using the *ab initio *motif finder program, DME. For each identified PWM, its quality was evaluated based on classification error rate calculated by the MOTIFCLASS program in CREAD package. In accordance with the classification error rates, PWMs were ranked and clustered so as to reduce redundancy (see Additional file 1). We used the highest ranked PWM in each cluster and added them to the *de novo *identified PWM set.

To identify TF binding motifs in promoters, we used the log odds ratio *L *between a PWM and background base frequency $f_{b}^{bg}$. We calculated log odds ratio *L*_*s *_for every subsequence of each promoter *s *(including the complementary strand), whose length is equal to the width of the motif of interest, *w*:

$$L_{s}=\sum_{i=1}^{w}\log\frac{f_{i}b_{i}}{f_{b_{i}}^{bg}}.$$

In our analyses, $f_{b}^{bg}$ is the base composition of each promoter, and the maximum of *L*_*s *_in a human promoter sequence was taken as the motif score *L*^*human *^for the sequence. For human genes whose mouse homologs are registered in Ensembl, *L*^*mouse *^is also calculated. Then, *L*^*human *^and *L*^*mouse *^were averaged to produce the final score *L*. We found that this incorporation of homologous regulatory information improves our results, while PWM search combined with an ordinary phylogenetic footprinting approach reduces the performance presumably owing to the loss of sensitivity. For human genes that do not have any homologs, we used *L*^*human *^as *L*. We assumed that the sequence has the motif if *L *is above the *p*% highest value in the population of all sequences. For all genes, we prepared binary data indicating the presence of the motif in their promoter with *p *= 5, 10, 15, and 20. This procedure was iterated for all members of the *de novo *identified and already known PWM set to produce the sequence feature table.

### Expression data analysis

Expression data [7] produced by Affymetrix GeneChips were downloaded from the Gene Expression Omnibus (GEO) database at NCBI (The GEO accession number is GSE3494). Absolute expression values of a data set were converted to the log scale and normalized so that the mean is equal to 0 and the variance is equal to 1 in each sample. The probe set IDs were converted to Ensembl gene IDs. In cases that one gene ID matches multiple probe set IDs, the probe set which shows the most variance among the samples was mapped to the gene. For in total 16,425 genes, we prepared meta-expression values for subsequent Bayesian network analysis by calculating differential expression between two sample groups or correlation with survival time as described below. The meta-expression values were also normalized so that the mean is equal to 0 and the variance is equal to 1.

Since the samples are separated into two groups, we measured differential expression of each gene between the two groups based on t-statistic. To evaluate the significance of differential expression, a null distribution of the t-statistic was produced from 100 data sets with randomly permutated sample labels. Based on the null distribution, the P-value was computed by two-sided test. To correct multiple hypotheses testing, the P-values were converted to Q-values using the qvalue package of R [23].

For Survival time information, we measured univariate correlation of each gene with survival time using the Cox proportional hazards regression method [24], we used the ratio of each regression coefficient to its standard error as the correlation value with poor prognosis.

### Bayesian network analysis

For selecting the network structure *N *of the Bayesian network, we apply a Bayesian approach. According to Bayes' theorem, the posterior probability of the network structure, *p*(*N*|*D*), is proportional to the product of the prior probability of the network structure, *p*(*N*) and the likelihood *p*(*D*|*N*) as

$$p(N|D)=\frac{p(N)p(D|N)}{p(D)}\propto p(N)p(D|N).$$

Based on this formula, we can infer the network structure *N *hidden behind the data *D*. In our analyses, we assumed that a network structure *N *is composed of a single child node and multiple parent nodes. The single child node has a continuous variable *x *representing a meta-expression value, and parent nodes have binary variables indicating the presence or absence of sequence features. The data *D *is composed of *M *meta-expression values and their sequence feature information. For a given data *D*, we search parent nodes, *i.e*., sequence features, for each group of meta-expressions by maximizing *p*(*N*|*D*).

#### The likelihood

Suppose that we have gene expression profiles of *M *genes measured by a number of microarrays. The meta-expression vector, *x*, is then computed as the *M*-dimensional vector whose the *i*th element, *x*_*i*_, represents the meta-expression value of the *i*th gene. We also assume that *S *is the sequence feature table whose the (*i*, *j*)th element, *s*_*ij*_, takes one if the *i*th gene has the *j*th sequence feature in its promoter region, or zero otherwise. The network structure, *N*, specifies the set of sequence features as the parents of the meta-expression values. For example, if *N *specifies the two parents for the meta expression values, we then consider a three nodes Bayesian network with observations $\left\{(x_{i},s_{ij1},s_{ij2}):i=1,...,M\right\}$, where *j*_1_, *j*_2 _∈ {1,..., *n*} and *j*_1 _≠ *j*_2_. Here *n *is the number of columns in *S*, *i.e*., the number of sequence features of interest. Our structural learning of Bayesian networks is to find the optimal combination of sequence features as the parents of meta-expression values.

In the problem stated above, we would like to discuss our model for meta-expressions when the networks structure is given. Since the information of sequence features take binary variables, *i.e*., 0 or 1, the parent variables can theoretically take $2^{n_{p}}$ patterns, where *n*_*p *_is the number of parents specified by the network structure. In the above example, the network model chooses two motifs as the parents and there are four patterns, {(0, 0), (0, 1), (1, 0), (1, 1)}, that the parents can take. In practice, since it is a possible case that we cannot find all the patterns of specified parents in *S *for large *n*_*p*_, we denote the number of observed patterns by *q *(≤ $2^{n_{p}}$). Therefore, if we specified the network structure, the meta-expression values can be separated into *q *exclusive groups. That is, the parents of the meta-expressions in each group show the same pattern.

More mathematically, let ***s***_*i *_= {*s*_*i*1_,..., *s*_*i*, *n*_} be the the *i*th row of *S*. Based on the specified structure *N*, we define the subset $s_{i}(N)=\{s_{i,{\text{p}}_{1}},...,s_{i,{\text{p}}_{r}}\}$ as the parents of meta-expressions, where {p_1_,...,p_*r*_} ⊂ {1,..., *n*}. We then have the following decomposition:

$$\begin{array}{lll} p(D|N) & = & \prod_{i=1}^{M}p(x_{i},s_{i}|N)\\ & = & \prod_{i=1}^{M}p(x_{i}|s_{i},N)p(s_{i}|N)\\ & \propto & \prod_{i=1}^{M}p(x_{i}|s_{i}(N))\\ & = & \prod_{k=1}^{q}p(d_{k}|pa_{k}), \end{array}$$

where ***pa***_*k *_is the *k*th pattern of parent motifs and ***d***_*k *_is the set of meta-expressions that have the same sequence feature information restricted by the parent motifs. For example, if ***s***_1_(*N*) and ***s***_2_(*N*) are equal to ***pa***_1_, then *x*_1 _and *x*_2 _are included in ***d***_1_. Note that we assume *p*(***s***_*i*_|*N*) = *p*(***s***_*i*_) follows uniform distribution and is independent from the selection of network structure *N*.

We next consider a statistical model for *p*(***d***_*k*_|***pa***_*k*_). By omitting the subscript *k *and the parent state, we denote *p*(***d***_*k*_|***pa***_*k*_) as *p*(***d***). Suppose that *M*_*k *_meta-expression values are included in the group, *i.e*., $d=\{x^{\left(1\right)},...,x^{\left(M_{k}\right)}\}$. Note that we also denote *M*_*k *_as *M *hereafter. We fit a normal distribution to each element of ***d ***by

$$\begin{array}{cc} \phi (x^{\left(m\right)}|\mu ,\tau )=\sqrt{\frac{\tau }{2\pi }}\exp\left\{-\frac{\tau {\left(x^{\left(m\right)}-\mu \right)}^{2}}{2}\right\}, & m=1,...,M, \end{array}$$

where *ϕ*(*x*|*μ*, *τ*) is the density of normal distribution with mean *μ *and variance *τ*^-1^. Note that *τ *is called precision. We assume that the joint prior density of mean and precision, *μ *and *τ*, is decomposed by

*p*(*μ*, *τ*) = *p*(*μ*|*τ*)*p*(*τ*).

The conditional density of *μ *is set as

$$p(\mu |\tau )=\phi (\mu |\mu_{0},\lambda_{0}\tau )=\sqrt{\frac{\lambda_{0}\tau }{2\pi }}\exp\left\{-\frac{\lambda_{0}\tau {\left(\mu -\mu_{0}\right)}^{2}}{2}\right\},$$

where *μ*_0 _and *λ*_0 _are hyperparameters. The marginal distribution of the precision, *τ*, is set by the density of gamma distribution with hyperparemeters, *α*_0 _and *β*_0_, and given by

$$p(\tau )=g(\tau |\alpha_{0},\beta_{0})=\frac{\beta_{0}^{\alpha_{0}}}{\Gamma (\alpha_{0})}\tau^{\alpha_{0}-1}\exp(-\beta_{0}\tau ).$$

In this setting, *p*(*μ*, *τ*) is the density of normal-gamma distribution with hyperparameters, *μ*_0_, *λ*_0_, *α*_0 _and *β*_0_. Hence, the marginal likelihood *p*(***d***) is given by

$$\begin{array}{lll} p(d) & = & \int_{\tau =0}^{\infty }\int_{\mu =-\infty }^{\infty }p(d|\mu ,\tau )p(\mu ,\tau )d\mu d\tau \\ & = & \int_{\tau =0}^{\infty }\int_{\mu =-\infty }^{\infty }\left\{\prod_{m=1}^{M}\phi (x^{\left(m\right)}|\mu ,\tau )\right\}\phi (\mu |\mu_{0},\lambda_{0}\tau )g(\tau |\alpha_{0},\beta_{0})d\mu d\tau . \end{array}$$

Since the normal-gamma distribution is a conjugate prior of normal distribution model, the integral in the marginal likelihood can analytically be calculated. Hence, by putting

$$\begin{array}{lll} \bar{x} & = & \frac{1}{M}\sum_{m=1}^{M}x^{\left(m\right)},\\ \lambda_{1} & = & \lambda_{0}+M,\\ \mu_{1} & = & \frac{\lambda_{0}\mu_{0}+M\bar{x}}{\lambda_{1}},\\ \alpha_{1} & = & \alpha_{0}+\frac{M}{2},\\ \beta_{1} & = & \beta_{0}+\frac{1}{2}\sum_{m=1}^{M}{\left(x^{\left(m\right)}-\bar{x}\right)}^{2}+\frac{M\lambda_{0}{\left(\bar{x}-\mu_{0}\right)}^{2}}{2\lambda_{1}}, \end{array}$$

we then have

$$p(d)=\frac{1}{{\left(2\pi \right)}^{M/2}}\cdot \frac{\Gamma (\alpha_{1})}{\Gamma (\alpha_{0})}\cdot \frac{\beta_{0}^{\alpha^{0}}}{\beta_{1}^{\alpha_{1}}}\cdot {\left(\frac{\lambda_{0}}{\lambda_{1}}\right)}^{1/2}.$$

The details of this calculation are shown in Additional file 1. Hence, the marginal likelihood, *p*(*D*|*N*), is obtained as the function of the hyperparameters {*μ*_0*j*_, *λ*_0*j*_, *α*_0*j*_, *β*_0*j*_} and is given by

$$p(D|N)=\prod_{k=1}^{q}\frac{1}{{\left(2\pi \right)}^{M_{k}/2}}\cdot \frac{\Gamma (\alpha_{1k})}{\Gamma (\alpha_{0k})}\cdot \frac{\beta_{0k}^{\alpha^{0k}}}{\beta_{1k}^{\alpha_{1k}}}\cdot {\left(\frac{\lambda_{0k}}{\lambda_{1k}}\right)}^{1/2}.$$

In our analysis, we set *μ*_0*k *_= 0, *λ*_0*k *_= 10, *α*_0*k *_= 9/2 and *β*_0*k *_= 10/2 for all *k*.

#### The prior probability

To avoid overfitting to the training data, the prior probability of the network *p*(*N*) was specified so as to penalize complex networks:

$$p(N)=cK^{-n_{p}},$$

where *c *is a constant that makes ∑*p*(*N*) = 1, *K *is a parameter that specifies how strongly complexity is penalized, and *n*_*p *_is the number of parent nodes in the network. As *K *decreases, the networks grow larger, and the number of parent nodes increases. Initially this increase in complexity reflects actual combinational regulation. However, after exceeding a point, false positive increase gradually owing to overfitting to the training data. To optimize the value of *K*, we performed preliminary runs with *K *= 10, 15, 20, 25, 30. We checked P-values for the training data, and chose *K *= 20 because it allows sufficient sensitivity and a minimum of false positives.

#### Search algorithm

To search for the most probable parent nodes based on the scoring function *p*(*N*)*p*(*D*|*N*), we took greedy search strategy. We started from structure without any edge between the child node and the parent node candidates and iteratively added an edge from a parent node candidate. For each iterative cycle, we calculated the score of *p*(*N*)*p*(*D*|*N*) for every case where the edge from the each parent node candidate was added, and the maximizer of them was added to the structure. The cycle repeated until no more edge increases the score. To speed up the search, we utilized clustering of parent node candidates (see Additional file 1).

## Results

### Transcriptional programs correlating with histological grades

Focusing on transcriptional regulatory programs that control histological diversity, we searched for *cis*-regulatory motifs associated with histological grades. Histological grading in breast cancer seeks to integrate measurements of cellular differentiation and replicative potential into a composite score that quantifies the aggressive behavior of a tumor. The most studied and widely used method is the Elston-Ellis modified Scarff, Bloom, Richardson grading system, also known as the Nottingham Grading System [25]. The Nottingham Grading System is based on a microscopic evaluation of morphologic and cytologic features of tumor cells, including degree of tubule formation, nuclear pleomorphism, and mitotic count. The sum of these scores stratifies breast tumors into grade 1 (G1; well-differentiated), grade 2 (G2; moderately differentiated), and grade 3 (G3; poorly diferentiated, highly proliferative) malignancies. It has been well known that the grade of breast cancer is a powerful indicator of disease recurrence and patient death. Untreated patients with G1 disease have a ~95% 5-year survival rate whereas those with G2 and G3 malignancy have survival rates at 5 years of ~75% and ~50%, respectively. Comparison between global expression profiles of tumor cells of different grades also revealed distinct expression patterns, especially between G1 and G3 groups [26].

**Figure 2:**
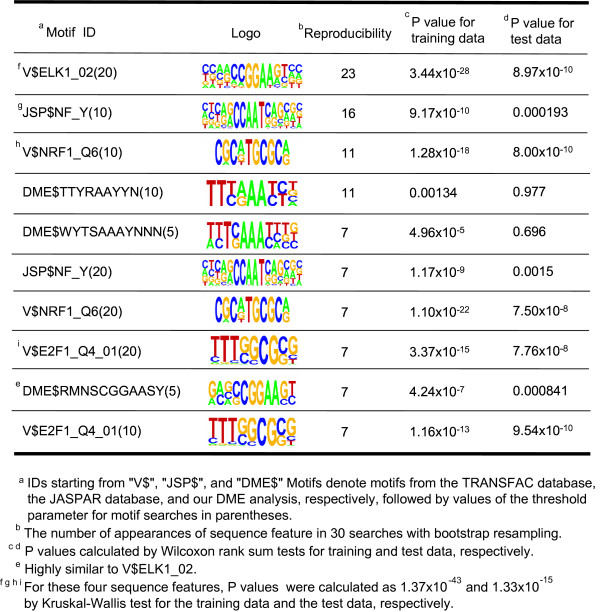
Sequence features associated with differential expression between G1 and G3 breast tumors.

For each gene in the global expression profile data, we calculated the degree of differential expression between two sample groups (67 G1 and 54 G3 samples). We then applied our method to the differential expression value to search for correlating motifs. The results were evaluated in two ways. First, reproducibility of the result was assessed by bootstrap analysis. Structure learning of a Bayesian network was repeated 30 times using bootstrap samples from the training dataset. We found that V$ELK1_02, V$E2F1_Q4_01, V$NRF1_Q6 and JSP$NF_Y were reproducibly selected by the bootstrap analysis (Figure 2). Here, IDs starting from "V$", "JSP$" and "DME$" motifs denote motifs from the TRANSFAC database, the JASPAR database and our DME analysis, respectively. For V$ELK1_02, highly similar motifs sampled by DME also reproducibly appeared. Although we present here results based on one training-test set partition, for checking robustness of biological findings, we applied our method to different training-test set partitions. We confirmed that almost the same results were obtained with different training-test set partitions. Secondly, statistical significance was evaluated for each of the sequence features reproducibly selected by the bootstrap analysis. We assessed difference of expression values between two gene groups with and without each sequence feature, using Wilcoxon rank sum test for the training and test data. It should be noted that, because the P-values calculated using the training data is not subject to multiple testing corrections, it can potentially achieve low values by overfitting to the training data. Hence, we must use the P-values calculated using the test data to accurately evaluate statistical significance. The results from the Wilcoxon rank sum tests suggest that sequence features that are most significantly associated with the histological grades are V$ELK1_02(20) V$E2F1_Q4_01(10), V$NRF1_Q6(10) and JSP$NF_Y(10) (The IDs are followed by values of the threshold parameter for motif searches in parentheses). P-values were also calculated for these four sequence features as a combination. We split genes into 16 groups based on combinations of the presence and absence of the 4 sequence feature, and evaluated difference of expression value distributions among the gene groups using Kruskal-Wallis test. Our calculation shows that the combination of these four sequence features scores highly significant a P-value of 1.33 × 10^-15 ^for the test data. Analyses using independent data sets and prediction based on the MAP-value also confirmed these results (see Additional file 1).

**Figure 3:**
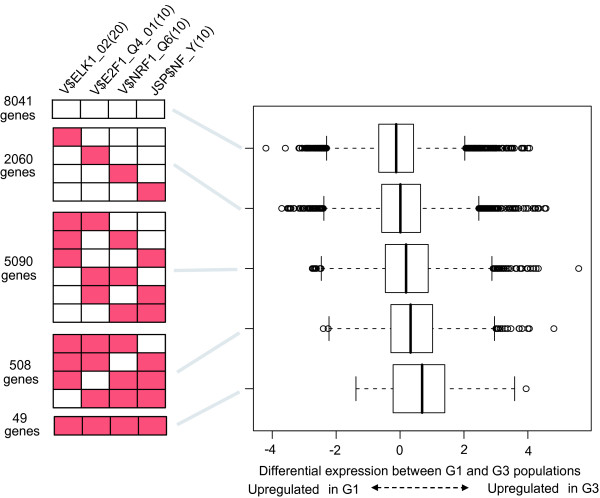
**Dependency of differential expression between G1 and G3 breast tumors on sequence features**. Genes are divided into five groups based on patterns of four sequence features, V$ELK1_02(20), V$E2F1_Q4_01(10), V$NRF1_Q6(10) and JSP$NF_Y(10) (the left red boxes indicates the presence of sequence features). The distributions of their differential expression values between G1 and G3 are displayed using box plots.

We next investigated how differential expression between G1 and G3 tumors depends on these four sequence features. We divided genes into 16 groups based on patterns of these four sequence features, and differences in distribution of their expression values were examined (see Supplementary Table 1 in Additional file 1). The box plots in Figure 3 summarize the results. For clarity, gene groups of similar distributions were gathered to form one group. These results indicate that these sequence features are additively associated with upregulation of gene expression in G3 populations.

**Table 1:** Motif associated with histological grades or prognosis identified based on independent datasets

	^*a*^Motif ID	^*b*^Reproducibility	^*c*^P value for training data	^*d*^P value for test data
	JSP$NF_Y(20)	20	3.1 × 10^-10^	0.000158
	V$NRF1_Q6(10)	15	3.09 × 10^-14^	6.02 × 10^-7^
motifs associated with histological grades based on the data by Sotiriou *et al*.	V$ELK1_02(20)	12	9.25 × 10^-26^	1.41 × 10^-6^
	DME$CTTCCGSYN(5)	9	5.71 × 10^-14^	6.82 × 10^-5^
	V$E2F1_Q4_01(5)	7	5.71 × 10^-15^	0.002372

	JSP$NF_Y(10)	15	2.46 × 10^-14^	0.011049
	DME$RMSYSSARGCGC(5)	11	4.02 × 10^-5^	0.063412
	V$ELK1_02(10)	10	2.03 × 10^-16^	2.08 × 10^-7^
motifs associated with prognosis based on the data by Sotiriou *et al*.	DME$YYYGSGCMYGCG(5)	8	1.65 × 10^-9^	0.008054
	V$E2F1_Q4_01(10)	8	1.05 × 10^-17^	2.37 × 10^-5^
	V$IRF_Q6_01(10)	7	2.06 × 10^-8^	0.000152
	DME$NMSTTCYKSYR(5)	6	0.000669	0.084446
	V$NRF1_Q6(20)	6	9.02 × 10^-22^	1.31 × 10^-6^

	JSP$NF_Y(20)	22	5.93 × 10^-8^	0.01116
motifs associated with histological grades based on the data by Pawitan *et al*.	V$E2F1_Q4_01(5)	10	6.56 × 10^-7^	0.049423
	DME$RCRKGCGCAVN(5)	6	5.71 × 10^-8^	0.060899
	V$E2F1_Q4_01(15)	6	9.59 × 10^-6^	0.017285

	V$ELK1_02(20)	16	1.26 × 10^-27^	6.13 × 10^-12^
	V$NRF1_Q6(15)	11	9.2 × 10^-23^	3.89 × 10^-7^
motifs associated with prognosis based on the data by Pawitan *et al*.	V$NRF1_Q6(20)	11	4.31 × 10^-22^	2.49 × 10^-7^
	V$ELK1_02(15)	9	1.63 × 10^-25^	6.41 × 10^-11^
	DME$RCGCHKGCGY(5)	6	3.23 × 10^-20^	4.8 × 10^-6^

^*a*^IDs starting from "V$", "JSP$", and "DME$" Motifs denote motifs from the TRANSFAC database, the JASPAR database, and our DME analysis, respectively, followed by values of the threshold parameter for motif searches in parentheses.

^*b*^The number of appearances of sequence feature in 30 searches with bootstrap resampling.

^*cd*^P values calculated by Wilcoxon rank sum tests for training and test data, respectively.

### Transcriptional programs correlating with prognosis

**Figure 4:**
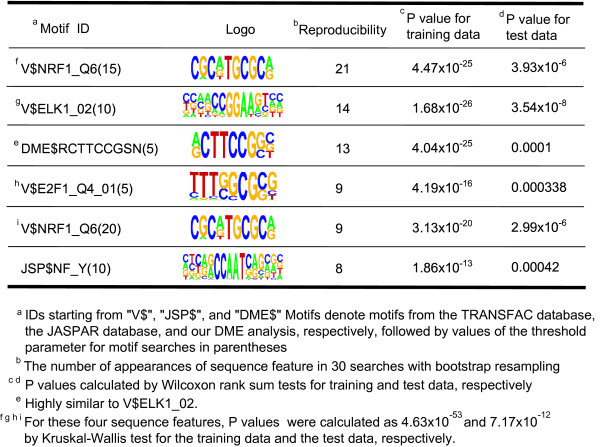
Sequence features associated with the correlation value calculated for breast cancer prognosis.

**Figure 5:**
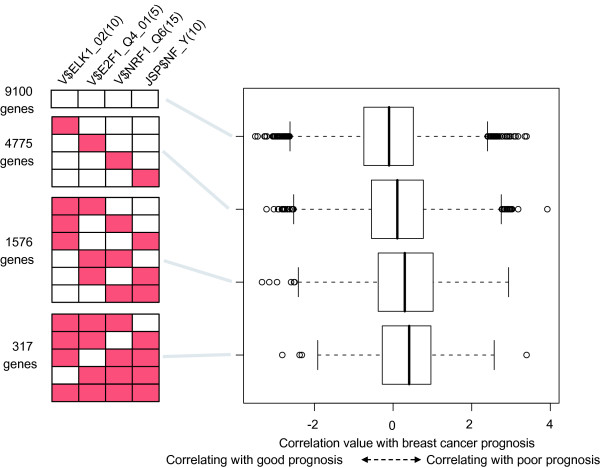
**Dependency of the correlation value with breast cancer prognosis on sequence features**. Genes are divided into five groups based on patterns of four sequence features, V$ELK1_02(5), V$E2F1_Q4_01(10), V$NRF1_Q6(15) and JSP$NF_Y(10) (the left red boxes indicates the presence of sequence features). The distributions of their correlation value with breast cancer prognosis are displayed using box plots.

We also examined regulatory programs associated with prognosis, a more direct measure of tumor malignancy. For each gene, correlation values with survival time were calculated using Cox regression models [24]. Then, we searched for *cis*-regulatory motifs associated with the correlation values using our method. Our analysis selected V$ELK1_02(10), V$E2F1_Q4_01(5), V$NRF1_Q6(15) and JSP$NF_Y(10) as sequence features positively associated with prognosis, similarly to the analysis for histological grade (Figure 4, Supplementary Table 2 in Additional file 1, and Figure 5). A P-value for a combination of these four motifs was calculated as 7.17 × 10^-12 ^for the test data.

### Robustness of our biological findings

To confirm robustness of our biological findings, we analyze independent data published by Sotiriou *et al*. [27](189 samples × 12466 genes) and Pawitan *et al*. [28](159 samples × 16425 genes). Similar to the results obtained in the above analyses, we found that the binding motifs of E2F, ELK1, NRF1 and NFY show significant correlation of histological grades and prognosis (Table 1), indicating the robustness of our findings. Taken together, we conclude that *cis*-regulatory motifs bound by these 4 TFs are principal motifs associated with breast cancer malignancy.

## Discussion

To decode transcriptional program in breast cancer, we developed a novel approach employing a new Bayesian scoring function and meta-expression value. Combining promoter sequence and expression data, we searched for *cis*-regulatory motifs correlated with histological grade and prognosis.

As motif sets to be searched, we prepared known motifs from databases, and *de novo *motifs identified by a motif discovery program, DME. As motifs correlated with malignancy, we identified the ELK1 binding motif as well as a highly similar *de novo *one, demonstrating success in our approach. Judging from statistical evaluations, the known motif shows better performance than the *de novo *one. Further improvement of the motif finder program will enable us to identify *de novo *motifs of higher quality. Our method introduced a new Bayesian approach, which can deal with multiple sequence features and a continuous meta-expression value. Compared to previous methods, our method more efficiently analyzes motif combination without thresholding meta-expression values (see Additional file 1). It should be noted that found motif combinations are no guarantee of a true synergistic, cooperative interaction of the related TFs; further studies remain to be done for analysis of motif interactions. Utilization of meta-expression values is also a novel feature of our method. Although we focused on histological grade and prognosis of breast cancer in this study, our approach can easily be extended to analyze other pathologies and other clinical variables. In addition to these features, we found that our method is robust on the data complexity; we found that our method leads to essentially the same result for grade-associated motifs even if we use only half of the patient data (see Supplementary Table 6 in Additional file 1).

Our analysis identified *cis*-regulatory motifs bound by ELK1, E2F1, NRF1 and NFY as principal motifs associated with breast cancer malignancy. ELK1 is a member of the ETS transcription factor family. Because the ETS family of transcription factors binds to similar motifs with a central core sequence GGA(A/T), ELK1 binding motifs are potentially bounded by other ETS family members. It has been reported that many of them are downstream nuclear targets of Ras-MAP kinase signaling, and the deregulation of the ETS genes results in malignant transformation and tumor progression. Several ETS genes are rearranged in human leukemia and Ewing tumor to generate chimeric oncoproteins. Furthermore, the aberrant expression of several ETS genes is often observed in various types of human malignant tumors [29]. Many of the ETS family transcription factors are upregulated in the G3 population: ETV7(*Q *= 7.79 × 10^-5^), ELF4(*Q *= 0.00182), ELF5(*Q *= 0.0270), GABPA(*Q *= 0.0301), SPIB(*Q *= 0.0344), ELF3(*Q *= 0.0383), ETV4(*Q *= 0.0386) and ETS1(*Q *= 0.0468). A recent study based on integrative bioinformatics also suggests that a ETS-directed transcriptional program is involved in malignant progression of prostate cancer [30]. Further integrative studies are required to examine whether ETS-directed transcriptional programs contributes to malignancy in various types of tumors.

The E2F family includes transcription factors which form heterodimer complexes with DP proteins and recognize a common motif [31]. The E2F family of proteins is known to be a master regulator of the cell cycle. The association of the E2F motif with G3 is therefore consistent with the fact that the histological grading criteria include the mitotic index and that G3 tumors are defined as highly proliferative. We also observed that most of the E2F family members and two DP genes are significantly upregulated in G3 tumors: E2F8(*Q *< 10^-6^), E2F3(*Q *< 10^-6^), E2F1(*Q *< 10^-6^), E2F6(*Q *= 3.14 × 10^-5^), E2F5(*Q *= 0.0219), DP2(*Q *= 0.00167) and DP1(*Q *= 0.0111).

NFR1 has been reported to induce nuclear-encoded mitochondrial genes and increase mitochondrial respiratory capacity [32]. Though no clear function of NRF1 in cancer cells has been reported, our finding that the NRF1-binding motif correlates with tumor malignancy may reflect hypermetabolism in aggressive tumors. It has also been reported that NRF1 collaborates with E2F family members to regulate genes involved in cellular proliferations [33].

The NFY-binding motif, the CCAAT box, is one of the first identified and most common elements in eukaryotic promoters. On the other hand, elucidation of regulatory networks involving NFY motifs has been hampered by their generality. Our result raises the possibility that NFY-binding motif functions malignant breast cancers cooperatively with other factors. In fact, a previous study reported that NFY and E2F functionally interact to regulate cell cycle genes [34].

Although we successfully identified above regulatory motifs, we failed to identify the motifs bounded by transcription factors that are thought to be more critically associated with breast cancer malignancy, including the estrogen receptor and p53. One reason for this failure is that, since the number of target genes varies between transcriptional regulators, our method "skims off" only strong signals from motifs bound by regulators having a sufficient number of target genes. However, a more likely reason is that our method focuses on only proximal regulatory sequences. Each TFs has a positional preference: some TFs bind mainly proximal promoters around the TSSs while others can act on distal enhancer sequences. Recent comprehensive ChIP analyses have clearly shown that the estrogen receptor and p53 have a broad range of positional preference [35,36]. Computational predictions [22] and genome-wide experiments [37,38] have just started to produce distal regulatory sequence data; incorporation of such information will solve this problem.

In cancer cells, genetic and epigenetic alterations also have great impact on gene expression at the mRNA level. Currently, comprehensive data of genomic copy number [39] and epigenetic status [40] are also accumulating. One of the next important challenges will be to incorporate them and decompose gene expression signals from different molecular mechanisms.

Considering the exploding availability of genome-wide experimental data, we can be optimistic that the integrative bioinformatics approach will circumvent these limitations in the near feature. Future work will focus on further refinement of our approach toward a deeper understanding of transcriptional programs in cancer cells.

## Conclusion

In this study, we introduced a new approach to analyze cancer microarray data. While many studies have focused on correlation between gene expression and a clinical phenotype, our method associates *cis*-regulatory motifs with clinical phenotypes. This approach offers a more concise description of transcriptome diversity among samples with different clinical phenotypes. Using this method, we demonstrated that *cis*-regulatory motifs bound by ELK1, E2F, NRF1 and NFY are most significantly associated with breast cancer malignancy. Our data suggest that they are principal regulatory motifs driving breast cancer malignant progression.

## Authors' contributions

AN, TA, ST, and HA designed research; AN performed research; AN, ADS and MQZ contributed new analytic tools; AN, TA, and SI wrote the paper.

## Supplementary Material

Additional file 1 **supplementary methods, discussions, tables and figures.** Click here for file
