# Supplementary material for: Integrative bioinformatics analysis of transcriptional regulatory programs in breast cancer cells
Source: BMC Bioinformatics. 2008 Sep 29;9:404. doi: 10.1186/1471-2105-9-404 (PMC2572072; doi:10.1186/1471-2105-9-404)

Supplementary Methods

Regulatory sequence analysis

Preparation of already known PWM set

As regulatory motif data, we prepared PWMs. The value f_{ib} of a PWM represents frequency of nucleotide base b at the i-th position in a motif. The frequencies of bases in each position are normalized so that $\sum_{b=a,t,g,c} f_{ib} = 1$. To avoid errors in log calculations, we reassigned 0.001 to f_{ib} equal to 0. we acquired a total of 495 PWMs, which consist of vertebrate 367 PWMs annotated as "good" in TRANSFAC 10.1 [4], 123 PWMs from JASPAR core [6], and 5 PWMs from original literature [3, 2]. We then removed extremely simple or complex PWMs and obtained a set of total 449 PWMs whose information contents range from 5 to 15. The information content R of a PWM is defined as follows:

$$R = 2w - \sum_{i=1}^{w} H_i,$$

where w is the width of the motif, and H_i is the information entropy at the i-th position defined by

$$H_i = - \sum_{b=a,c,g,t} f_{ib} \log_2 f_{ib}.$$

Since this set includes highly redundant PWMs, they were subjected to clustering to reduce the redundancy.

For clustering, the dissimilarity between two PWMs A and B was calculated based on the Kullback-Leibler divergence. At every alignment offset, the PWMs were extended using a column representing the uniform base frequency ($f_{ib} = 0.25$ for all b) so that all positions of two aligned motifs were matched. For every pair of the extended PWMs, A' and B', whose length are w', the dissimilarity $D_{A'B'}$ is calculated by:

$$D_{A'B'} = \sum_{i=1}^{w'} \sum_{b=a,t,g,c} (f_{ib}^{A'} - f_{ib}^{B'}) \log \frac{f_{ib}^{A'}}{f_{ib}^{B'}}.$$

We assumed the lowest score of $D_{A'B'}$ as the dissimilarity between A and B, D_{AB}. Note that $D_{AB} = D_{BA}$ holds.

Using the partition around medoids algorithm in the R package with the dissimilarity criterion, the 449 PWMs are divided into 250 clusters. We used 250 medoids of the clusters as the already known PWM set in the following analyses.

Preparation of de novo identified PWM set

In addition to the already known PWM set prepared by the above procedure, we prepared PWMs overrepresented in promoter sequence of genes with high or low values in the expression value data. For the top 500 and the bottom 500 genes for expression values in the training data, we obtained their promoter sequences (the 500bp upstream and the 100bp downstream of the TSS) and those of their mouse homologs. Then, we searched the two groups of sequences for motifs overrepresented in either group using the ab initio motif finder program, DME [5].

Given foreground (FG) and background (BG) sequence sets and the base composition f of $FG \cup BG$, DME iteratively identifies the top motif M, ranked according to the ratio $L_{FG,P}(M,f)/L_{BG,Q}(M,f)$ of maximum likelihood scores, where $L_{S,Z}(M,f)$ is the likelihood of M and base composition f, given sequence set S with values for the missing data Z maximizing the scoring function:

$$L_{F,Z}(M,f) = \prod_{s_i \in F} Pr(s_i|M)^{z_i} Pr(s_i|f)^{(1-z_i)}.$$

DME searches were performed against two reverse combinations of foreground and background sequences with a variety of parameter settings. The parameters specify number of motifs output (n), width size (w), granularity (g), refinement (r), and average information (i), and were set as follows: (n, w, g, r, i); (30, 8, 0, 0.125, 1.8); (30, 9, 0, 0.125, 1.675); (30, 10, 0.5, 0.125, 1.6); (30, 11, 1.0, 0.125, 1.575); (30, 1.0, 0.5, 0.25, 1.55). Then, for each identified PWM, its quality was evaluated based on classification error rates calculated by the MOTIFCLASS program in CREAD package (downloaded from http://rulai.cshl.edu/cread/). The classification error rates were based on the maximum scoring subsequence in a given promoter and threshold set to minimize this error. In accordance with the classification error-rates, PWMs were ranked and clustered to reduce redundancy. In the ranked motif list, the first motif in the rank was assumed as the representative of the first cluster. From the second motif, every motif was visited in the order of decreasing rank, and the dissimilarities from the first motif were calculated as described above. If the dissimilarity is below a threshold (10 in our study), the motif was assigned to the first cluster. After removing members of the first cluster from the ranked list, the same procedure was repeated for the remainder until all motifs formed clusters. We used the highest ranked PWM in each cluster as a member of a de novo identified PWM set in the following analyses.

Visualization of PWMs

For graphical presentations of PWMs, we produced sequence logos using the open source code of the Weblogo program (downloaded from http://weblogo.berkeley.edu/) [1].

Bayesian network analysis

The likelihood (deduction of the marginal probability)

The marginal likelihood $p(\boldsymbol{d})$ is given by

$$\begin{aligned} p(\boldsymbol{d}) &= \int_{\tau=0}^{\infty}\int_{\mu=-\infty}^{\infty} p(\boldsymbol{d}|\mu,\tau)p(\mu,\tau)d\mu d\tau \\ &= \int_{\tau=0}^{\infty}\int_{\mu=-\infty}^{\infty} \left\{\prod_{m=1}^{M}\phi(x^{(m)}|\mu,\tau)\right\}\phi(\mu|\mu_0,\lambda_0\tau)g(\tau|\alpha_0,\beta_0)d\mu d\tau. \end{aligned}$$

If we define

$$\begin{aligned} \bar{x} &= \frac{1}{M}\sum_{m=1}^{M} x^{(m)}, \\ \lambda_1 &= \lambda_0 + M, \\ \mu_1 &= \frac{\lambda_0\mu_0 + M\bar{x}}{\lambda_1}, \\ \alpha_1 &= \alpha_0 + \frac{M}{2}, \\ \beta_1 &= \beta_0 + \frac{1}{2}\sum_{m=1}^{M}(x^{(m)}-\bar{x})^2 + \frac{M\lambda_0(\bar{x}-\mu_0)^2}{2\lambda_1}, \end{aligned}$$

some simple algebra can show that

$$\sum_{m=1}^{M}(x^{(m)}-\mu)^2 + \lambda_0(\mu-\mu_0)^2 = \lambda_1(\mu-\mu_1)^2 + \sum_{m=1}^{M}(x^{(m)}-\bar{x})^2 + \frac{M\lambda_0(\bar{x}-\mu_0)^2}{\lambda_1},$$

which means that we can now rewrite the marginal probability as:

$$p(\boldsymbol{d}) = \frac{\lambda_0^{1/2}}{(2\pi)^{M/2}}\frac{\beta_0^{\alpha_0}}{\Gamma(\alpha_0)}\iint (2\pi)^{-1/2}\tau^{1/2}\exp[-0.5\lambda_1\tau(\mu-\mu_1)^2]\tau^{\alpha_1-1}\exp[-\beta_1\tau]\,d\mu\,d\tau.$$

Note that quantity inside the integral is proportional to a Normal-Gamma distribution with parameters $\{\mu_1,\lambda_1,\alpha_1,\beta_1\}$

$$\iint (2\pi)^{-1/2}(\lambda_1\tau)^{1/2}\exp[-0.5\lambda_1\tau(\mu-\mu_1)^2]\frac{\beta_1^{\alpha_1}}{\Gamma(\alpha_1)}\tau^{\alpha_1-1}\exp[-\beta_1\tau]\,d\mu\,d\tau = 1.$$

Using this, we can rewrite the marginal probability as:

$$p(\boldsymbol{d}) = \frac{\lambda_0^{1/2}}{(2\pi)^{M/2}} \cdot \frac{\beta_0^{\alpha_0}}{\Gamma(\alpha_0)} \cdot \frac{\Gamma(\alpha_1)}{\beta_1^{\alpha_1}\lambda_1^{1/2}} = \frac{1}{(2\pi)^{M/2}} \cdot \frac{\Gamma(\alpha_1)}{\Gamma(\alpha_0)} \cdot \frac{\beta_0^{\alpha_0}}{\beta_1^{\alpha_1}} \cdot \left(\frac{\lambda_0}{\lambda_1}\right)^{1/2}.$$

Search algorithm

It is computationally infeasible to search network structures exhaustively for the one maximizing the score of $p(N)p(D|N)$ except when the number of nodes is small. In our analyses, to search combinations of hundreds of parent node candidates, we took a greedy search strategy. The data D is composed of M observations (corresponding to genes) for n parent candidate variables (corresponding to sequence features) and one child variable. Some candidate variables correspond to sequence features made from an identical PWM with different threshold values. Because such variables show distributions similar to each other, we clustered them to one group before structure learning. Starting from a structure without any edge between the child node and the parent node candidates, we iteratively added an edge from a parent node candidate. For each iterative cycle, we calculated the score of $p(N)p(D|N)$ for every case that the edge from the representative node of each cluster was added. Next, we chose clusters whose score ranked among the top 20. After all members belonging to the 20 cluster were scored similarly, the maximizer of them was added to the structure. The cycle repeated until no more edge increases the score. To further reduce computational time, we restricted the search space at and after the second iteration to clusters whose centers increase the score at the first iterative cycle. After the greedy search, the combination of parent nodes was optimized. Within the cluster of each selected parent node, the parent node was replaced with another member which increases $p(N)p(D|N)$, if such a node exists. This step was repeated for the cluster of every parent node until a round of these steps did not change the combination of parent nodes.

A pseudocode for this algorithm is as follows:

Problem: maximize the score of $p(N)p(D|N)$
Inputs: data D composed of M observation for n parent candidate variables and one child variable, clusters of parent candidate variables
Outputs: a combination of parent nodes N

find_parent_nodes(data D, clusters of parent candidate variables)
{
  do{

```
    for(each cluster){
      calculate p(N)p(D|N) for the case that the representative node is add
      if(the score is within top 20){
        record the cluster as the top 20 clusters
      }
    }
    for(each member of the top 20 clusters){
      calculate p(N)p(D|N) for the case that the node is add
      if(the score is the maximum){
        record the node as the maximizer
      }
    }
    add the maximizer to N
  }until(no more node increases p(N)p(D|N))
  return a combination of parent nodes N
}
```

Supplementary Discussions

Comparison to a previous method

Several studies have reported integrative analyses similar to our analysis. For example, Rhodes *et al.* [7] analyzed cancer transcriptional programs based on gene sets called "signatures". First they obtained two types of gene sets; gene sets that show differential expression between different types of cancers as "expression signatures", and genes that have a common *cis*-regulatory motif as "regulatory signatures". Significance of the overlap between these two types of signatures then can be evaluated based on the hypergeometric distribution. They reported significant pairs of expression and regulatory signatures as functional transcriptional programs. To compare our method to another method, we searched for *cis*-regulatory motif correlating with histological grades according to their method. Because appropriate thresholds are needed in their method, we prepared signatures using multiple threshold parameters. To prepare expression signatures, we sorted genes based on differential expression between G3 and G1 tumors, and obtained the 1, 3, 10, and 30 % top-ranked genes. For regulatory signatures, we prepared gene sets that posses each motif assuming multiple PWM thresholds. For each pair of two types of signature, we calculated the number of genes in the expression signature, e, the number of genes in the regulatory signature, r, the size of

the overlap between two signatures, o, and the total number of genes, t. A P-value is then calculated as follow:

$$P = \sum_{i=o}^{\min(e,r)} \frac{{}_{(t-r)}C_{(e-i)} \cdot {}_{r}C_{i}}{{}_{t}C_{e}}.$$

Table 5 shows the 20 top-ranked sequences which show the lowest P-values. Similarly to our method, the binding motifs of E2F, ELK1, NRF1, and NFY show significant P-values, when some threshold values were applied (Note that multiple testing corrections are furthermore necessary in this approach). However, not all threshold values lead to significant results, suggesting optimization of the parameters is critical. On the other hand, our method does not need such threshold optimization. Furthermore, while their method analyzes motifs individually, our method can analyze multiple motifs simultaneously as a combination of motifs.

Prediction based on the MAP (maximum a posteriori) value

The MAP (maximum a posteriori) value is defined as follows:

$$\hat{\theta}_{MAP} = \arg\max_{\theta}(p(\theta|D, N)).$$

To predict meta-expression values in the test data, we use the MAP value of μ_k. When a combination of parent nodes N is specified, Data D can be divided into q groups according to parent patterns. μ_k is a parameter specifying the mean of the expression values whose parent pattern belongs to the kth group. In our model, μ_{1k} corresponds to the MAP value of μ_k. Using the training data, we calculated μ_{1k} based on the parent patterns of the 4 significant sequence features. We then predict meta-expression values of the test data from their parent patterns and the MAP value of μ_k. Figures 1 and 2 demonstrate correlations between predicted observed and predicted values for histological grades and prognosis. Significance tests for Pearson's correlation shows highly significant P values of $< 2.2 \times 10^{-16}$ and 6.439×10^{-15}, respectively. These result confirmed that the binding motifs of E2F, ELK1, NRF1, and NFY are strongly associated with breast cancer malignancy.

References

[1] Crooks, G. E., Hon, G., Chandonia, J. M. and Brenner, S. E. (2004). WebLogo: a sequence logo generator, *Genome Res*, **14**, 1188-1190.

[2] Hallikas, O., Palin, K., Sinjushina, N., Rautiainen, R., Partanen, J., Ukkonen, E. and Taipale, J. (2006). Genome-wide prediction of mammalian enhancers based on analysis of transcription-factor binding affinity, *Cell*, **124**, 47-59.

[3] Loh, Y. H., Wu, Q., Chew, J. L., Vega, V. B., Zhang, W., Chen, X., Bourque, G., George, J., Leong, B., Liu, J., Wong, K. Y., Sung, K. W., Lee, C. W., Zhao, X. D., Chiu, K. P., Lipovich, L., Kuznetsov, V. A., Robson, P., Stanton, L. W., Wei, C. L., Ruan, Y., Lim, B. and Ng, H. H. (2006). The Oct4 and Nanog transcription network regulates pluripotency in mouse embryonic stem cells, *Nat Genet*, **38**, 431-440.

[4] Matys, V., Kel-Margoulis, O. V., Fricke, E., Liebich, I., Land, S., Barre-Dirrie, A., Reuter, I., Chekmenev, D., Krull, M., Hornischer, K., Voss, N., Stegmaier, P., Lewicki-Potapov, B., Saxel, H., Kel, A. E. and Wingender, E. (2006). TRANSFAC and its module TRANSCompel: transcriptional gene regulation in eukaryotes, *Nucleic Acids Res*, **34**, D108-D110.

[5] Smith, A. D., Sumazin, P. and Zhang, M. Q. (2005). Identifying tissue-selective transcription factor binding sites in vertebrate promoters, *Proc Natl Acad Sci U S A*, **102**, 1560-1565.

[6] Vlieghe, D., Sandelin, A., De Bleser, P. J., Vleminckx, K., Wasserman, W. W., van Roy, F. and Lenhard, B. (2006). A new generation of JASPAR, the open-access repository for transcription factor binding site profiles, *Nucleic Acids Res*, **34**, D95-D97.

[7] Rhodes, D. R., Kalyana-Sundaram, S., Mahavisno, V., Barrette, T. R., Ghosh, D., Chinnaiyan, A. M. (2005). Mining for regulatory programs in the cancer transcriptome, *Nat Genet*, **37**, 579-583

Supplementary Tables and Figures

Table 1: Dependency of differential expression between G1 and G3 breast tumors on sequence features. The training and test data were divided into 16 groups based on patterns of 4 sequence features, V$ELK1_02(20) V$E2F1_Q4_01(10), V$NRF1_Q6(10) and JSP$NF_Y(10). For each group, the count of genes, the mean and the standard deviation (SD) of the differential expression values between G1 and G3 breast tumors are displayed.

V$E2F1_Q4_01 (10)	V$ELK1_02 (20)	V$NRF1_Q6 (10)	JSP$NF_Y (10)	In training data			In test data		
				count	mean	SD	count	mean	SD
0	0	0	0	6028	-0.125	0.924	2013	-0.13	0.927
0	0	0	1	735	-0.00952	1.06	229	0.075	1.14
0	0	1	0	717	0.043	1.01	256	0.0977	0.981
0	0	1	1	119	0.217	1.17	38	0.162	1.22
0	1	0	0	1781	0.0857	0.997	578	0.0283	0.973
0	1	0	1	299	0.292	1.09	109	0.149	1.02
0	1	1	0	426	0.207	0.973	118	0.264	1.04
0	1	1	1	64	0.587	1.18	37	0.222	0.884
1	0	0	0	597	0.0312	1.05	197	0.00691	1.02
1	0	0	1	175	0.19	1.16	65	0.359	1.33
1	0	1	0	196	0.32	1.09	60	0.119	1.12
1	0	1	1	70	0.448	1.06	28	0.413	0.888
1	1	0	0	343	0.233	1.12	112	0.389	1.07
1	1	0	1	85	0.507	1.1	34	0.233	1.12
1	1	1	0	137	0.346	1.13	53	0.62	0.985
1	1	1	1	34	0.629	1.24	15	0.897	1.21

Table 2: Dependency of the correlation value with breast cancer prognosis on sequence features. The training and test data were divided into 16 groups based on patterns of 4 sequence features, V$ELK1_02(5) V$E2F1_Q4_01(10), V$NRF1_Q6(15) and JSP$NF_Y(10). For each group, the count of genes, the mean and the standard deviation (SD) of the correlation values with breast cancer prognosis are displayed.

V$E2F1_Q4_01 (5)	V$ELK1_02 (10)	V$NRF1_Q6 (15)	JSP$NF_Y (10)	In training data			In test data		
				count	mean	SD	count	mean	SD
0	0	0	0	6854	-0.121	0.972	2246	-0.108	0.973
0	0	0	1	861	0.0754	1.03	303	0.0469	1
0	0	1	0	1475	0.0725	0.992	536	0.0927	1.02
0	0	1	1	293	0.306	1.03	90	0.267	1.08
0	1	0	0	895	0.176	0.994	284	0.194	0.959
0	1	0	1	151	0.246	1.04	63	0.168	1.02
0	1	1	0	383	0.299	1.02	102	0.183	1.03
0	1	1	1	70	0.357	0.925	21	0.193	1.23
1	0	0	0	300	0.122	0.997	101	0.169	1.05
1	0	0	1	111	0.227	1.09	31	0.642	1.01
1	0	1	0	177	0.344	0.942	58	0.202	0.963
1	0	1	1	54	0.665	1	30	0.0128	0.994
1	1	0	0	86	0.382	1.08	31	0.489	0.805
1	1	0	1	28	0.552	0.761	11	0.165	0.939
1	1	1	0	61	0.413	0.941	23	0.0902	0.97
1	1	1	1	16	0.256	0.819	3	-0.0887	0.394

Table 3: Bootstrap analysis for motifs associated with histological grades. IDs in each row represent motifs selected in each trial in the bootstrap sample.

V$ELK1_02$20	V$NRF1_Q6$10	JSPNF_Y10	DME$TTYRAAYYN$15	
V$ELK1_02$20	V$NRF1_Q6$10	JSPNF_Y10	DME$TTYRAAYYN$15	
V$ELK1_02$20	V$NRF1_Q6$10	JSPNF_Y10	DME$TTYRAAYYN$15	
V$ELK1_02$20	V$NRF1_Q6$10	JSPNF_Y10	DME$TTYRAAYYN$15	
V$ELK1_02$20	V$NRF1_Q6$10	JSPNF_Y10	DME$TTYRAAYYN$15	
V$ELK1_02$20	V$NRF1_Q6$10	JSPNF_Y10	DME$TTYRAAYYN$15	
V$ELK1_02$20	V$NRF1_Q6$10	JSPNF_Y10	DME$TTYRAAYYN$15	
V$ELK1_02$20	V$NRF1_Q6$10	JSPNF_Y10	DME$TTYRAAYYN$15	
V$ELK1_02$20	V$NRF1_Q6$10	JSPNF_Y10	DME$TTYRAAYYN$15	
V$ELK1_02$20	V$NRF1_Q6$10	JSPNF_Y10	DME$TTYRAAYYN$15	
V$E2F1_Q4_01$20	V$ELK1_02$20	V$NRF1_Q6$10	DME$TTYRAAYYN$15	DME$GRDRRSARA$10
V$ELK1_02$20	JSPNF_Y10	DME$MCCGCCCWSNM$5		
V$ELK1_02$20	JSPNF_Y10	DME$MCCGCCCWSNM$5		
V$ELK1_02$20	JSPNF_Y10	DME$MCCGCCCWSNM$5		
V$E2F1_Q4_01$20	V$ELK1_02$20	DME$SYRCGCMKGCKC$5	DME$WYTSAAAYNNN$5	
V$E2F1_Q4_01$20	V$ELK1_02$20	DME$SYRCGCMKGCKC$5	DME$WYTSAAAYNNN$5	
V$E2F1_Q4_01$20	V$ELK1_02$20	DME$SYRCGCMKGCKC$5	DME$WYTSAAAYNNN$5	
V$E2F1_Q4_01$20	V$ELK1_02$20	DME$SYRCGCMKGCKC$5	DME$WYTSAAAYNNN$5	
V$ELK1_02$20	JSPNF_Y10	DME$MCCGCCCWSNM$5		
V$E2F1_Q4_01$20	V$ELK1_02$20	DME$SYRCGCMKGCKC$5	DME$WYTSAAAYNNN$5	
V$ELK1_02$20	JSPNF_Y10	DME$MCCGCCCWSNM$5		
V$ELK1_02$20	JSPNF_Y10	DME$MCCGCCCWSNM$5		
V$E2F1_Q4_01$20	V$ELK1_02$20	DME$SYRCGCMKGCKC$5	DME$WYTSAAAYNNN$5	
V$E2F1_Q4_01$10	V$NRF1_Q6$20	JSPNF_Y20	DME$RMNSCGGAASY$5	DME$WYTSAAAYNNN$10
V$E2F1_Q4_01$10	V$NRF1_Q6$20	JSPNF_Y20	DME$RMNSCGGAASY$5	DME$WYTSAAAYNNN$10
V$E2F1_Q4_01$10	V$NRF1_Q6$20	JSPNF_Y20	DME$RMNSCGGAASY$5	DME$WYTSAAAYNNN$10
V$E2F1_Q4_01$10	V$NRF1_Q6$20	JSPNF_Y20	DME$RMNSCGGAASY$5	DME$WYTSAAAYNNN$10
V$E2F1_Q4_01$10	V$NRF1_Q6$20	JSPNF_Y20	DME$RMNSCGGAASY$5	DME$WYTSAAAYNNN$10
V$E2F1_Q4_01$10	V$NRF1_Q6$20	JSPNF_Y20	DME$RMNSCGGAASY$5	DME$WYTSAAAYNNN$10

Table 4: Bootstrap analysis for motifs associated with prognosis. IDs in each row represent motifs selected in each trial in the bootstrap sample.

V$NRF1_Q6$15	JSPNF_Y10	DME$RCTTCCGSN$5	
V$ELK1_02$10	V$NRF1_Q6$15	DME$TKTWNCCWN$10	DME$NRRCCAATV$10
V$ELK1_02$10	V$NRF1_Q6$15	DME$TKTWNCCWN$10	DME$NRRCCAATV$10
V$NRF1_Q6$15	DME$RCTTCCGSN$5		
V$NRF1_Q6$15	DME$RCTTCCGSN$5		
V$NRF1_Q6$15	DME$RCTTCCGSN$5		
V$NRF1_Q6$15	DME$RCTTCCGSN$5		
V$NRF1_Q6$15	DME$RCTTCCGSN$5		
V$NRF1_Q6$15	DME$RCTTCCGSN$5		
V$NRF1_Q6$15	DME$RCTTCCGSN$5		
V$NRF1_Q6$15	DME$RCTTCCGSN$5		
V$NRF1_Q6$15	DME$RCTTCCGSN$5		
V$NRF1_Q6$15	DME$RCTTCCGSN$5		
V$NRF1_Q6$15	DME$RCTTCCGSN$5		
V$NRF1_Q6$15	JSPNF_Y10	DME$RCTTCCGSN$5	
V$NRF1_Q6$15	JSPNF_Y10	DME$RCTTCCGSN$5	
V$NRF1_Q6$15	JSPNF_Y10	DME$RCTTCCGSN$5	
V$E2F1_Q4_01$5	V$ELK1_02$10	V$NRF1_Q6$20	
V$E2F1_Q4_01$5	V$ELK1_02$10	V$NRF1_Q6$20	
V$E2F1_Q4_01$5	V$ELK1_02$10	V$NRF1_Q6$20	
V$E2F1_Q4_01$5	V$ELK1_02$10	V$NRF1_Q6$20	
V$E2F1_Q4_01$5	V$ELK1_02$10	V$NRF1_Q6$20	
V$E2F1_Q4_01$5	V$ELK1_02$10	V$NRF1_Q6$20	
V$E2F1_Q4_01$5	V$ELK1_02$10	V$NRF1_Q6$20	
V$E2F1_Q4_01$5	V$ELK1_02$10	V$NRF1_Q6$20	
V$E2F1_Q4_01$5	V$ELK1_02$10	V$NRF1_Q6$20	
V$NRF1_Q6$15	JSPNF_Y10	DME$RCTTCCGSN$5	

Table 5: Motif search based on signatures. Regulatory signatures were prepared based on sequence features in our method. Expression signatures are composed of the 1, 3, 10, and 30 % most upregulated in G3 tumor compared to G1 tumors. For each overlap between two types of signatures, P-values were calculated using the hypergeometric distribution.

	1%	3%	10%	30%
V$E2F1_Q4_01(5)	2.05×10^{-11}	1.39×10^{-11}	4.55×10^{-13}	0.223
V$E2F4DP1_01(5)	1.88×10^{-8}	4.79×10^{-8}	1.38x10–11	0.964
V$ELK1_02(5)	0.708	0.0127	8.54×10^{-11}	0.236
V$E2F4DP1_01(10)	5.41×10^{-7}	9.01×10^{-7}	1.13×10^{-10}	0.381
V$NRF1_Q6(5)	0.0505	2.30×10^{-6}	1.65×10^{-10}	6.84×10^{-5}
JSP$NF_Y(5)	3.86×10^{-9}	4.63×10^{-10}	1.29×10^{-9}	0.992
V$E2F1_Q4_01(10)	4.93×10^{-10}	9.86×10^{-10}	1.94×10^{-9}	0.177
V$ELK1_02(10)	0.568	0.00469	1.22×10^{-8}	0.0114
JSP$NF_Y(10)	2.82×10^{-8}	4.02×10^{-7}	1.78×10^{-5}	0.992
V$NRF1_Q6(10)	0.0164	5.80×10^{-5}	2.98×10^{-8}	0.000223
V$NRF2_01(5)	0.833	0.0101	1.13×10^{-7}	0.983
V$E2F1_Q3_01(10)	0.00525	0.00165	8.40×10^{-7}	0.477
V$E2F1_Q3_01(5)	0.0288	0.00852	2.56×10^{-6}	0.949
V$NRF1_Q6(15)	0.204	0.0359	0.00411	7.68×10^{-6}
V$E2F1_Q4_01(15)	1.24×10^{-5}	2.52×10^{-5}	2.75×10^{-5}	0.000393
V$E2F4DP1_01(15)	1.94×10^{-5}	0.00121	0.00123	0.00128
V$NRF1_Q6(20)	0.541	0.545	0.716	5.24×10^{-5}
V$ACAAT_B(5)	0.000174	0.000863	0.00048	0.999
JSP$NF_Y(15)	0.000229	0.00217	0.687	0.999
V$E2F1_Q4_01(20)	0.000951	0.0392	0.0821	0.0593

Table 6: Motif associated with histological grades identified based on only half of the patient data.

[a] Motif ID	[b] Reproducibility	[c] P value for training data	[d] P value for test data
V$ELK1_02$20	24	1.12E-41	3.03E-10
[e] DME$YDBYNATTGG$10	9	0.00018	0.005364
V$NRF1_Q6$15	8	2.00E-25	1.52E-14
V$E2F4DP1_01$15	6	1.53E-17	0.000632
JSPNF_YS20	6	1.99E-09	0.001144

[a] IDs starting from "V$", "JSP$", and "DME$" Motifs denote motifs from the TRANSFAC database, the JASPAR database, and our DME analysis, respectively, followed by values of the threshold parameter for motif searches in parentheses.

[b] The number of appearances of sequence feature in 30 searches with bootstrap resampling.

[c d] P values calculated by Wilcoxon rank sum tests for training and test data, respectively.

[e] Similar to JSP$NF_Y

Figure 1: Prediction of differential expression values between G3 and G1 tumor. Differential expression values in the test data were predicted from 4 sequence features (V$ELK1_02(20) V$E2F1_Q4_01(10), V$NRF1_Q6(10), and JSP$NF_Y(10)) and the MAP value of μ_k learned from the training data. Correlation between observed and predicted values scores a Spearman correlation coefficient of 0.1396 and a P value of $< 2.2 \times 10^{-16}$.

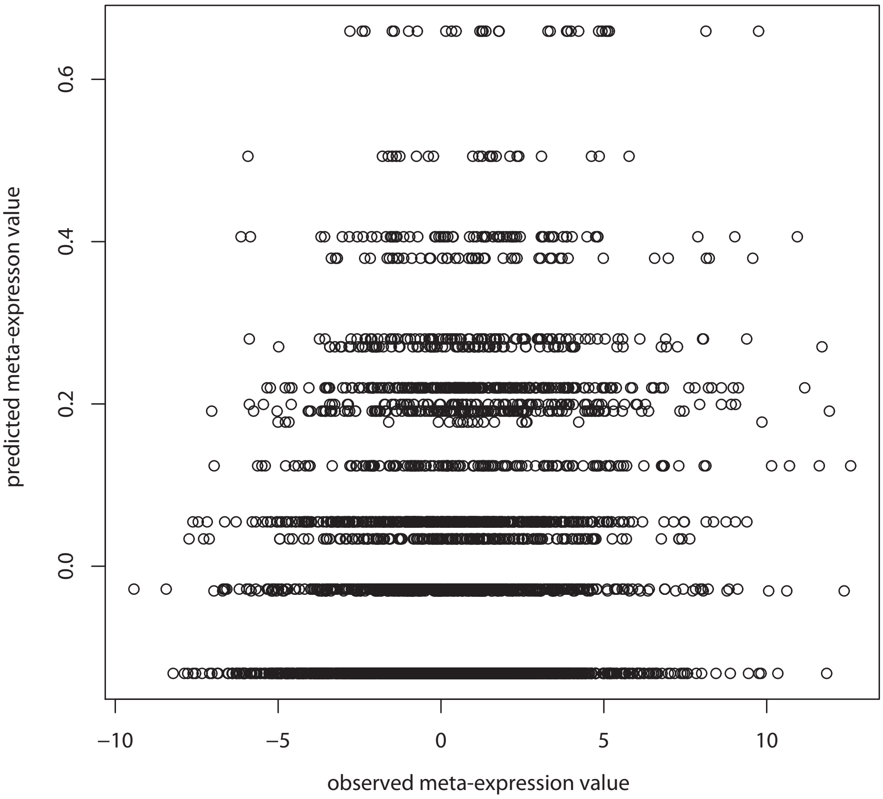

Figure 2: Prediction of prognosis correlation values. Prognosis correlation values in the test data were predicted from 4 sequence features (V$ELK1_02(5) V$E2F1_Q4_01(10), V$NRF1_Q6(15), and JSP$NF_Y(10)) and the MAP value of μ_k learned from the training data. Correlation between observed and predicted values scores a Spearman correlation coefficient of 0.1355 and a P value of $< 2.2 \times 10^{-16}$.

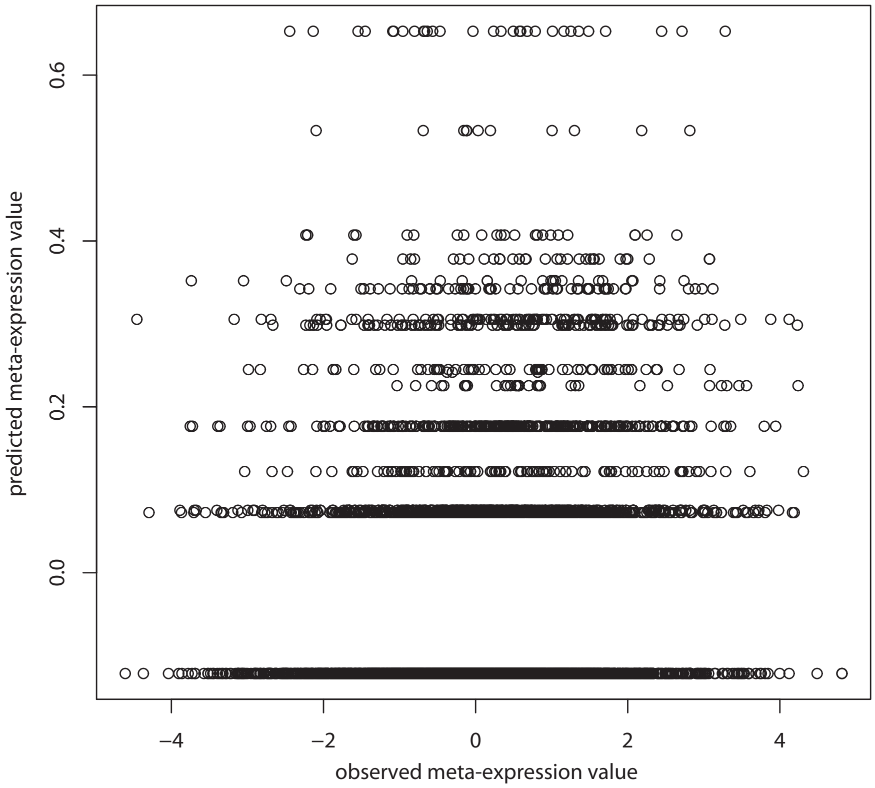

Supplement: Additional file 1 — supplementary methods, discussions, tables and figures. [file 1471-2105-9-404-S1.pdf]
